# Letter from Dr. Smelfungus

**Published:** 1855-12

**Authors:** S. Smelfungus


*Letter from Dr. Smelfungus.*—It is with no small pleasure that we give place to the following letter from our ancient friend, Dr. Smelfungus:

Editor Buffalo Med. Jour.

Dear Sir: The recent discussion of the subject of pneumonia in the Medical Association of your city, is my apology for breaking my long silence, and trotting myself out as a party in the argument. Please turn to page 666, of vol. viii., of your monthly, and find my views, and "when found make a note of it." Speaking of the tendency of pneumonia to self-limitation, I there remark that "As Ollapod says, 'hence we view,' that you do not in every case cut short the disease with your routine of bloodletting, calomel, antimony, and blistering. *It cuts itself short*, and therein only manifests its natural tendency."

So much for the natural history of the disease, and now for my views as to treatment, which I believe to be "excelled by few and equalled by none," in point of pith and brevity:

"Shall we, then, abandon bloodletting in pneumonia? Even so; for it is generally unnecessary. Not so; for cases there be when the delirious mind grows rational, the swollen countenance natural, and the choked and labored pulse grows soft and easy, from the lancet.

"Shall we abandon calomel and antimony? Again yes; and again no; for cases will occur when every means that science can prompt or art direct, are necessary to guide and govern the lava-tide of inflammation, to prevent effusion and abscess and the whole dark array of sequelæ."

"And blistering;" and Smelfungus speaks tenderly as a lover of his mistress: "blisters are always good, and never disappoint us." If in all the nauseous scented armamentaria of physic there is one thing that Smelfungus is willing (metaphorically speaking) to take to his heart, it is Emplastrum Cantharidis! He loves his blisters as fervently as old Dr. Clysterpipe his syringes, for the old man based his claim to Christian character *on the love he bore his enemas (enemies!)*

"Listen, then, to the truthful lesson! Pneumonia is still, and ever will be, a disease eminently requiring the guiding hand of the physician. It is, in a majority of cases, a self-limited and little dangerous disease; but it should be closely watched, lest, as sometimes happens, the diseased action may not stop at the usual point in the lower lobe, but rage on unchecked throughout its utmost borders. And mark you, man of the lancet! He who cures a pneumonia predestined to occupy a whole lung, does a goodly thing, and may congratulate himself. Here come in your whole catalogue of remedies. The God Antiphlogos alone is mighty to save!"

After mature reflection I am convinced that the sentiments above quoted are grounded in the sure word of prophecy. But, gentlemen of the association! *Perge! Go ahead!* Your discussions are moderate in tone but weighty in argument. Out of your much discourse shall come order and settled views. As for me—*inveni portum!*

*Valete.*

S. SMELFUNGUS, M. D.

---

*The Pronouncing Medical Lexicon, containing the Correct Pronunciation and Definition of most of the terms used by Speakers and Writers on Medicine and the Collateral Sciences.* With Addenda. By C. H. CLEAVELAND, M. D., Member of the American Medical Association, of the American Association for the Advancement of Science, Professor of Materia Medica and Therapeutics in the E. M. Institute, &c., &c. Cincinnati: Longley Brothers. 1855.

The "E. M. Institute" mentioned in the title-page as honored by the author's holding in it a "professorship," is the Eclectic Medical Institute of Cincinnati. "Eclectic" means to select, to choose from, and C. H. Cleaveland, M. D., is a true eclectic, inasmuch as he has * * * * * * (not knowing what word to use we substitute sta s) the whole of this little book from Reese's Medical Lexicon, only impairing its value by changing the title-page, and interpolating a "phonetic pronunciation." We recommend its purchase to our readers if they will but buy it under the old title of Reese's Medical Lexicon.

